# Sickle cell disease patients with COVID‐19 in Guadeloupe: Surprisingly favorable outcomes

**DOI:** 10.1002/jha2.449

**Published:** 2022-05-04

**Authors:** Emmanuelle Bernit, Marc Romana, Scylia Alexis‐Fardini, Vanessa Tarer, Pierre‐Marie Roger, Lydia Doumdo, Eléonore Petras, Corine Charneau, Benoit Tressières, Marie Dominique Hardy Dessources, Maryse Etienne‐Julan

**Affiliations:** ^1^ Sickle Cell Disease Unit Reference Centre for Sickle Cell Disease Thalassemia and Other Red Cell Rare Diseases CHU de la Guadeloupe Pointe‐à‐Pitre Guadeloupe France; ^2^ Laboratoire d'Excellence du Globule Rouge (Labex GR‐Ex) PRES Sorbonne Paris France; ^3^ Université des Antilles UMR_S1134/Inserm BIGR Pointe‐à‐Pitre Guadeloupe France; ^4^ Université de Paris UMR_S1134 BIGR INSERM Paris France; ^5^ Service d'infectiologie CHU de la Guadeloupe Pointe à Pitre Guadeloupe France; ^6^ Sickle Cell Disease Unit Centre Hospitalier de la Basse‐Terre Basse‐Terre France; ^7^ Centre d'Investigation Clinique Antilles Guyane Inserm CIC Point‐à‐Pitre Guadeloupe France

**Keywords:** Coronavirus disease 2019, COVID‐19, French West Indies, pneumonia, SARS‐CoV2 virus, sickle cell disease

## Abstract

We investigate risk factors for hospitalization and difference between sickle cell syndromes in a cohort of COVID‐19 sickle cell disease (SCD) adult patients managed in the Reference Center of Guadeloupe. We retrospectively collected data of symptomatic SCD adult patients infected with SARS‐CoV‐2 between March and December 2020. Thirty‐eight SCD adult patients with symptomatic COVID‐19 infection were included during the first wave, representing 9.6% of the active patient file at our center. The median age (IQR) was 39 years (24–47). Four patients were obese and two had moderate renal failure. The median duration of symptoms (IQR) was 10 days (5–15). Seventeen (44.7%) patients were hospitalized, including two in intensive care unit (ICU) for acute chest syndrome. An 85‐year‐old SC patient with prostate cancer died. No difference was detected between inpatient and outpatient groups in terms of age, gender, BMI, SCD clinical complications, and in history SCD treatment. There was no difference for severity, hospitalization, length of stay, ICU stay, or death between SS or Sβ°‐thal patients and SC or Sβ^+^‐thal patients. These overall favorable outcomes among symptomatic patients may be related to the low prevalence of comorbidity known to be linked to the more severe forms of COVID‐19, but also to the prompt coordinated management of SCD patients in the Reference Center.

**Abbreviations**
ACE: angiotensin‐converting enzyme
ACS: acute chest syndrome
BMI: body‐mass index
CI: confidence interval
CK‐EPI: chronic kidney disease epidemiology collaboration
COVID‐19: Coronavirus disease 2019
CRP: C‐reactive protein
CT scan: computed tomography scan
et al.: et alter
GFR: glomerular filtration rate
Hb: hemoglobin
Hg: mercury
HU: hydroxyurea
ICU: intensive care unit
IQR: interquartile range
kg: kilogram
mg: milligram
mm: millimetre
pO_2_
: pressure of oxygen
RBCs: red blood cells
RCU: red cell units
RR: relative risk
RT‐PCR: reverse transcription‐polymerase chain reaction
SARS‐CoV‐2: severe acute respiratory syndrome‐coronavirus 2
SCD: Sickle cell disease
SpO_2_
: estimate of oxygen saturation
thal: thalassemia
UK: United Kingdom
US: United States
VOC: vaso‐occlusive crisis
WHO: World Health Organization

## 1 INTRODUCTION

Sickle cell disease (SCD) is an inherited disorder of hemoglobin (Hb) that predominantly involves individuals of African descent, affecting millions of people in sub‐Saharan Africa [1]. In Guadeloupe, a French West Indies Island of 379,710 inhabitants in 2020 of which about 90% are of Afro‐Caribbean origin, SCD is the most common genetic disease affecting 1 in 300 newborns, and 11% of the population carries an abnormal β globin gene [2].

To manage SCD patients, a Reference Center has been created within the University Hospital of Guadeloupe since 1990. SCD results from the synthesis of the abnormal Hb S that polymerizes in deoxygenated conditions, leading to the sickling of red blood cells (RBCs). Sickle RBCs are more rigid, fragile, and therefore prone to disruption [3]. While homozygous HbS disease (SS) is the most common type of SCD and considered as the most severe sickle syndrome, less common types of SCD arise as a result of double heterozygosity between HbS gene and different β‐globin gene mutations such as Hb C (SC) or β‐thalassemia (Sβ‐thal) that share some similar basic pathophysiology processes leading to variable phenotypes. Clinically, SCD is characterized by chronic hemolytic anemia and vaso‐occlusive events resulting from abnormal interactions between abnormal RBCs, white blood cells, platelets, and endothelial cells, leading to painful acute vaso‐occlusive crisis (VOC), acute chest syndrome (ACS), stroke, and a broad range of acute and chronic complications affecting every organ system [3]. Patients with SCD have increased susceptibility to infections, which is partly due to autosplenectomy resulting from recurrent vaso‐occlusive infarcts within the spleen, but involvement of leukocytes functions, cell‐mediated immunity, antibody production, alternate complement pathway, and abnormalities of opsonization have also been reported [4, 5, 6].

Several concerns were raised with the pandemic spread of the severe acute respiratory syndrome‐coronavirus 2 (SARS‐CoV‐2). The impact of various viruses on SCD clinical course has been described. It is already known that influenza and dengue outbreaks cause excess morbidity in SCD population who experience increased VOC events and ACS with an increased need for intensive care and exchange transfusions during these viral infections [7, 8, 9, 10]. In addition, SC patients, although exhibiting less severe SCD clinical course, have a higher rate of severe dengue fever and death than those with SS genotype [9, 11]. Therefore, SCD patients are considered to be at increased risk of COVID‐19 complications with a higher risk of life‐threatening ACS. This assumption is based on pulmonary tropism of the virus, impaired immunity and systemic vasculopathy of SCD patients that predispose them to end organ dysfunction, and high risk of thrombosis [12].

Data on poor outcome of COVID‐19 in adults with SCD have been published through small cohorts from France, England, or America [13, 14, 15, 16]. More recently, risk factors for hospitalization and serious COVID‐19 in SCD patients have been reported in 750 children and adults with SCD and COVID‐19 illness registered in the international SECURE‐SCD Registry [17]. Despite these reports, the impact of SARS‐CoV‐2 infection in SCD patients still raises questions: is SCD a risk factor for severe COVID‐19? Does this viral infection induce VOC and ACS events and the need for intensive care? Are the different characteristics of sickle cell patients and their comorbidities associated with different prognoses? We report here characteristics and prognosis of Guadeloupean adult patients infected by SARS‐CoV2 during 2020 first and second waves and managed in the same center by investigating risk factors for hospitalization and by evaluating possible differences between SS and SC patients.

## 2 METHODS

### 2.1 Study population

This retrospective study was conducted at the Reference Center for SCD of Guadeloupe, located in University Hospital of Guadeloupe. In 2020, 394 SCD patients aged 16 and over and living in this French archipelago were seen at least once during the year and were followed up regularly.

We identified patients who presented to the Reference Center or emergency unit of the University Hospital with clinical symptoms consistent with SARS‐CoV‐2 infection and for which a COVID‐19 diagnosis was established by reverse transcription‐polymerase chain reaction (RT‐PCR) test of a nasopharyngeal sample and/or COVID‐19 serological analysis without any other differential diagnosis. Search for dengue infection was also systematically performed because dengue outbreak was occurring at the same period. All patients were followed up clinically after diagnosis for 6–12 months. None of them were lost to follow up.

There was no systematic screening in our center, thus patients with asymptomatic COVID‐19 were not identified. Therefore, the incidence of SARS‐CoV‐2 infection in the SCD population of Guadeloupe could not be computed.

### 2.2 Data collection and clinical definition

In March 2021, we retrospectively collected in our reference center data of symptomatic adult SCD patients infected with SARS‐CoV‐2 between March and April 2020 for the first wave and between August and December 2020 for the second wave. In accordance with the declaration of Helsinki, the project was approved by the local Research Ethics Committee and written consent was obtained from each subject. Data were anonymized prior to statistical analysis.

#### 2.2.1 SCD clinical and biological features

Demographic information, genotype, medical history including medical treatment, were collected. The basal level of Hb was obtained at least 2 months after or before any acute complication of the SCD and up to 3 months before presentation with COVID‐19 or after blood transfusion. Severe occlusive disease in the previous 3 years was defined by more than two hospitalized VOC or priapism and/or by more than one ACS during this period. VOC was diagnosed as hyperalgesic if pain level was higher than 6 on 10 by numeric pain scale and required high opioid doses. ACS is defined as a new pulmonary infiltrate and some combination of fever, chest pain, and signs and symptoms of pulmonary diseases such as tachypnea, cough, and dyspnea [18].

Co‐morbidities known to impact severity of SCD and SARS‐CoV‐2 infection were reported. Glomerular filtration rate (GFR) was estimated by using chronic kidney disease epidemiology collaboration (CKD‐EPI) [19]. Moderate renal failure corresponds to a reduction of GFR between 45 and 59 ml/min/m^2^. According to the WHO definition, a body mass index (BMI) equal or greater than 25 is overweight and a BMI equal or greater than 30 is obesity. Mellitus diabetes was reported if the patient was treated with insulin and/or oral antidiabetic drugs. High blood pressure >13/8 define arterial hypertension [20]. Cardiac insufficiency was defined by heart disease requiring specific treatment. Pulmonary hypertension was defined as a mean pulmonary artery pressure >20 mm Hg on right heart catheterization. Pulmonary obstructive disease was diagnosed on pulmonary function tests. Cerebral vasculopathy was reported if the patient had an abnormal transcranial Doppler and/or cerebral imaging and/or if the patient had a history of transient cerebral attack or stroke or cerebral hemorrhage.

#### 2.2.2 SARS‐CoV‐2 clinical and biological features

The following infectious symptoms were noted during consultations and/or hospitalization: fever ≥38.5°C, viral symptoms (chills, arthromyalgia, asthenia), respiratory symptoms (rhinitis, cough, dyspnea), oxygen saturation lower than 95%, tachycardia, agueusia, anosmia, gastrointestinal signs (abdominal pain, diarrhea, vomiting), and headache. Median duration of symptoms, outpatient or inpatient care, treatments during infection, laboratory tests and hospitalization length of stay for inpatients, need for intensive care, and mortality were also collected. Criteria for hospitalization were saturation <94%, respiratory rate >24/min, worsening anemia >2 points, VOC requiring opioids, ACS, neurological signs or digestive signs suggesting dehydration. SARS‐CoV‐2 infection was defined as severe if there was hypoxemia <92%, ACS, hyperalgesic VOC or if blood transfusion was required. COVID‐19 pneumonia was diagnosed on chest scan (at least if there was the presence of ground grass opacities) with injection or not according to renal function. Individual patient's hypoxemia was diagnosed by pulse oximeter placed on fingers (SpO_2_) or by determining the oxygen level in blood gas sample (pO_2_). Oxygen was supplied to maintain SpO_2_ higher than 97% and respiratory rate <24/min. Lowest value of Hb, highest value of neutrophils, lowest value of lymphocytes, and highest values of creatinine and C‐reactive protein (CRP), are collected for each patient within 2 weeks following the COVID‐19 diagnosis.

### 2.3 Statistical analysis

Quantitative variables were summarized as mean with standard deviation (SD) or median with interquartile range (IQR) depending on their distribution and compared across groups using Student *t*‐test or Mann–Whitney non‐parametric test, respectively. Categorical data were expressed as percentages and compared between groups using Chi‐square test or Fisher exact test, depending on the sample size. Clinical history, gender, age, genotype, clinical and biological data of inpatients and outpatients were compared. Clinical features and outcome of SARS‐CoV‐2 acute infection in SS or Sβ^0^‐thal (SS/Sβ^0^‐thal) patients were also compared to those of SC and Sβ^+^‐thal (SC/Sβ^+^‐thal) patients. All statistical analyses were performed using R 4.1.0. Significance was considered at the level 5%.

## 3 RESULTS

### 3.1 Demographic and clinical characteristics

Thirty‐eight adult SCD patients with either confirmed or probable COVID‐19 infection were included in this study (Table 1). Nasal COVID‐19 PCR was performed in 28 cases (74%) and was positive in 26/28 cases. The two patients whose RT‐PCR was negative and the 10 patients whose RT‐PCR could not be performed exhibited a suggestive clinical history, a positive COVID 19 serological test, and no differential diagnosis.

**TABLE 1:** Demographic and clinical characteristics and basal hemoglobin level of the adult sickle cell cohort infected with SARS‐CoV‐2 in Guadeloupe

		**Cohort**	**SC/Sβ+**	**SS/Sβ0**	
***N***		**38**	**14/1**	**22/1**	***p***
Age	Median [Q25–Q75]	39 [24–47]	39 [35–47]	39 [25–48]	0.893
Sex ratio	M / F	14 / 24	4 / 11	10 / 13	0.480
BMI	Median [Q25–Q75]	22.3 [19.5–26.5]	25.8 [22.1–28.5]	20.3 [18.5–23.6]	**0.004**
Basal Hb (g/dl)	Median [Q25–Q75]	9.5 [8.0–10.6]	11.0 [10.5– 11.5]	8.5 [7.2– 9.5]	**<0.001**
**Comorbid conditions**					
Chronic kidney disease	*n* (%)	10 (27.0)	5 (35.7)	5 (21.7)	0.585
Renal insufficiency	*n* (%)	2 (5.4)	2 (14.3)	0 (0.0)	0.137
Cardiac insufficiency	*n* (%)	2 (5.4)	1 (7.1)	1 (4.3)	1.000
Obstructive pulmonary disease	*n* (%)	3 (8.1)	0 (0.0)	3 (13.0)	0.135
Cerebral vasculopathy	*n* (%)	5 (13.5)	0 (0.0)	5 (21.7)	0.135
**Severe occlusive disease in the last 3 years**					
>2 hospitalized VOC/priapism	*n* (%)	4 (11.4)	0 (0.0)	4 (0.0)	0.119
>1 ACS	*n* (%)	7 (20.0)	1 (6.7)	6 (30.0)	0.199
**Treatment, *n* (%)**					
Hydroxyurea	*n* (%)	15 (39.5)	1 (6.7)	14 (60.9)	**0.002**
Exchange transfusion program	*n* (%)	2 (5,4)	1 (7.1)	1 (4.3)	1.000
Transfusion's program	*n* (%)	11 (29.7)	2 (14.3)	9 (39.1)	0.150
Phlebotomy's program	*n* (%)	5 (13.5)	5 (35.7)	0 (0.0)	**0.005**
ACE inhibitor	*n* (%)	7 (18.9)	3 (21.4)	4 (17.4)	1.000
Home oxygen therapy	*n* (%)	14 (36.8)	2 (13.3)	12 (52.2)	**0.020**

Abbreviations: ACS, acute chest syndrome; ACE inhibitor, angiotensin‐converting enzyme inhibitor; BMI, body mass index; Hb, hemoglobin; VOC, vaso‐occlusive crisis. *p*‐Values compare **SC/Sβ+** with **SS/Sβ0** patients. Bold *p*‐values indicate statistically significant results (*p* < 0.05).

Thirty‐eight symptomatic patients were included, that is, 9.6% of the active file of the SCD center. Twenty‐two patients had SS disease, 14 SC disease, one Sβ^0^‐thal, and one Sβ^+^‐thal disease. Table 1 shows the demographic, clinical, and biological characteristics of the cohort. The median age was 39 years [IQR, 24–47]. Five patients were overweight and three were obese. None of the included patients had diabetes mellitus or pulmonary hypertension. Two SC patients had moderate renal failure. The patients with cerebral vasculopathy had been stabilized for several years and were no longer on exchange transfusions. Five patients were treated with a single or a combination therapy for hypertension. One patient was on an anticoagulant for a history of thromboembolic disease. Eleven patients had presented severe occlusive disease in the previous 3 years (Table 1). One patient was 15 weeks pregnant at the time of infection. As expected, basal Hb was significantly lower in the SS/ Sβ^0^‐thal group that also includes significantly more patients under hydroxyurea (HU) treatment and home oxygen therapy (Table 1).

### 3.2 Clinical features related to COVID‐19

The most frequently reported infectious clinical features were cough (57%), fever (49%), and arthromyalgia (40%). Only one (2.6%) patient had pulmonary embolism. The median time from the onset of symptoms to the start of management was 2 days (IQR [1–9]). The median duration of viral symptoms was 10 days (IQR [5–15]).

### 3.3 Clinical and biological characteristic, management and outcome of SCD patients

Table 2 shows antecedents, SCD clinical features, and biological characteristics of inpatients and outpatients. Seventeen patients were hospitalized (44.7%), including two in intensive care unit (ICU; 1 SC and 1 SS) for ACS without true hemodynamic or respiratory criteria but for close monitoring. None of them required mechanical ventilation. The duration in ICU was 3 and 4 days, respectively. An 85‐year‐old SC patient managed for prostate cancer for one year unfortunately died with VOC and mesenteric ischemia without pulmonary symptoms at day 5 of hospitalization. The median time from symptom onset to hospitalization was 1.5 days (IQR [0.3–8.5]). For these 17 patients hospitalized, SCD complications—VOC, ACS, or worsening anemia—were the cause of hospitalization in 10 patients (58.8%). Only one SC woman with ACS experienced pulmonary embolism. During their hospitalization, all patients received oxygen therapy, 10 patients (58.8%) received VOC treatment with intravenous hydration and analgesia, and 6 were managed for ACS (35.3%). Of the 12 chest scans performed on respiratory signs, only 4 showed specific lesions of COVID‐19 pneumonia. Chest CT scan showed one or more parenchymal condensations in favor of ACS in four patients and chest CT was normal in four patients. No patient was co‐infected with dengue virus.

**TABLE 2:** Clinical characteristic, SCD complications, and biological features of inpatients and outpatients of the adult sickle cell cohort infected with SARS‐CoV‐2 in Guadeloupe

		**Outpatients**	**Inpatients**	
***N* (%)**		**21 (55)**	**17 (45)**	***p***
Age	median [Q25–Q75]	42 [27–49]	31 [24–46]	0.347
Sex ratio	M/F	0.61	0.54	
BMI	Median [Q25–Q75]	22.3 [20.8‐26.5]	21.1 [18.4‐26.5]	0.244
Basal Hb (g/dl)	Median [Q25–Q75]	9.4 [7.3–11.0]	9.5 [8.8–10.3]	0.531
**Genotype**				
SS/Sβ0	*n* (%)	13 (61.9)	10 (58.8)	0.999
SC/Sβ+	*n* (%)	8 (38.1)	7 (41.2)	1.000
**Comorbid conditions**				
Arterial hypertension	*n* (%)	5 (23.8)	0 (0.0)	0.107
Nephropathy	*n* (%)	8 (38.1)	2 (12.5)	0.173
Respiratory disease	*n* (%)	2 (9.5)	1 (6.2)	1.000
Cardiac disease	*n* (%)	1 (4.8)	1 (6.2)	1.000
Cerebral vasculopathy	*n* (%)	4 (19.0)	1 (6.2)	0.52
**Treatment**				
Hydroxyurea	*n* (%)	6 (28.6)	9 (53.9)	0.232
ACE inhibitor	*n* (%)	7 (33.3)	0 (0.0)	**0.032**
**Severe disease in the last 3 years**				
>2 hospitalized VOC/priapism	*n* (%)	3 (14.3)	1 (7.1)	0.914
>1 ACS	*n* (%)	4 (19.0)	3 (21.4)	1.000
**SCD clinical features**				
VOC	*n* (%)	2 (9.5)	10 (58.8)	**0.004**
ACS	*n* (%)	0 (0.0)	6 (35.3)	**0.012**
**Laboratory values**				
Lowest value of Hb (g/dl)	Median [Q25–Q75]	9.4 [7.8–10.5]	8.6 [6.7–9.9]	0.323
Highest value of neutrophils (G/L)	median [Q25–Q75]	3.7 [3.0–5.9]	8.3 [5.6–11.1]	**0.003**
Lowest value of lymphocytes (G/L)	Median [Q25–Q75]	2.0 [1.5–3.2]	1.7 [0.8–2.7]	0.275
Highest value of creatinine (μmol/l)	Median [Q25–Q75]	56.5 [53.5–64.5]	60.5 [45.0–72.3]	0.853
Highest value of CRP (mg/L)	Median [Q25–Q75]	11.0 [4.9–17.3]	71.0 [40.0–202.0]	**0.005**

Abbreviations: ACE inhibitor, angiotensin‐converting enzyme inhibitor; ACS, acute chest syndrome; BMI, body mass index; CRP, C‐reactive protein; Hb, hemoglobin; VOC, vaso‐occlusive crisis. The values for Hb, neutrophils, lymphocytes, creatinine, and CRP are collected for each patient within 2 weeks of the COVID‐19 diagnosis. Bold *p*‐values indicate statistically significant results (*p* < 0.05).

Only one SC patient, hospitalized for a hyperalgesic VOC that rapidly evolved into ACS despite several iterative phlebotomies for Hb level higher than 14 g/dl, benefited from a short corticosteroid therapy over 3 days at 1 mg/kg with a rapid improvement of the respiratory symptoms and without secondary complication after corticosteroid therapy. No other patient received specific COVID‐19 therapy. Three patients (2 SS, 1 SC) received transfusions (2 red cell units (RCU), 3 RCU and 2 RCU respectively); two patients (SS and SC) received exchange transfusions (2 RCU and 3 RCU) in the ICU.

The median length of hospitalization was 5 days (IQR [3–8]). A patient on HU treatment for stabilized cerebral vasculopathy presented several episodes of severe VOC ± ACS in the 3 months after his COVID‐19 episode, requiring transfusion exchange program resumption still on going. Except this patient and the elderly patient who died, all others recovered without sequelae.

Among the 21 adults who were not hospitalized (Table 2), only two of them presented a mild VOC (9.5%) associated with infectious signs, requiring the prescription of analgesics. Eight patients received home oxygen therapy. Among these outpatients, only one 64‐year‐old female SC patient complained of intense asthenia compatible with a long‐COVID‐19 for 5 months after infectious episode with complete recovery at 6 months.

### 3.4 Comparison between inpatients and outpatients

No difference was detected between inpatient and outpatient groups in terms of age, gender, BMI, SCD clinical complications studied as well as in terms of SCD severity of vaso‐occlusive complications developed in the previous 3 years (Table 2). There was no difference in history of SCD treatment provided, specific infectious symptoms presented, duration of symptoms or time‐delay from symptom onset up to management. However, inpatients had significantly higher neutrophils count median and higher value of CRP than outpatients (Table 2).

It is worth noting that seven outpatients (33%) but none of inpatients were treated by on angiotensin‐converting enzyme (ACE) inhibitors and that hospitalized patients had significantly more VOC and ACS during their COVID‐19 than non‐hospitalized patients (Table 2).

### 3.5 Comparison between SS or Sβ^0^‐thal patients and SC or Sβ^+^‐thal patients

Table 3 shows comparison between patients with SS or Sβ^0^‐thal disease and those with SC or Sβ^+^‐thal disease. Despite the expected differences for BMI and basal Hb value (Table 1), there was no difference in terms of severity, hospitalization and length of stay, ICU stay, or death between the two groups.

**TABLE 3:** Clinical features, management and outcome of the adult SC/Sβ^+^‐thal and SS/Sβ^0^‐thal patients infected with SARC‐CoV‐2 in Guadeloupe

		**SC/Sβ+**	**SS/Sβ0**	
***N***		**15**	**23**	***P***
**Clinical features**				
Oxygen saturation <94%	*n* (%)	1 (6.7)	8 (36.4)	0.094
Tachycardia	*n* (%)	5 (33.3)	1 (4.5)	0.060
VOC	*n* (%)	4 (26.7)	8 (34.8)	0.866
ACS	*n* (%)	2 (13.3)	4 (17.4)	1.00
Pulmonary embolism	*n* (%)	1 (6.7)	0 (0.0)	0.827
**Outcome**				
Days of symptoms	Median [Q25–Q75]	14 [6–18]	9.5 [5–14]	0.199
Hospitalization	*n* (%)	7 (46.7)	10 (43.5)	1.000
ICU	*n* (%)	1 (6.7)	1 (4.3)	1.000
Hospitalization days	Median [Q25–Q75]	4 [2–7]	6 [3–8]	0.322
Death	*n* (%)	1 (6.7)	0 (0.0)	0.395
Recovery without sequelae	*n* (%)	14 (93.3)	22 (95.5)	1.000

Abbreviations: ACE inhibitor, angiotensin‐converting enzyme inhibitor; ACS, acute chest syndrome; BMI, body mass index; ICU, intensive care unit; VOC, vaso‐occlusive crisis.

## 4 DISCUSSION

We present here the impact of COVID‐19 disease in a cohort of 38 SCD adults composed mainly of SS (57%) and SC (37%) patients. There has been a prompt management of the COVID‐19 infection for inpatients and outpatients and median of symptoms duration did not exceed 14 days. Only four patients with respiratory symptoms had specific pneumonia of COVID‐19 and none of them required mechanical ventilation. However, 32% of our cohort exhibited VOC and 16% experienced ACS during COVID‐19. Nearly 30% (29.4%) of inpatients received simple or exchange transfusion because of vaso‐occlusive complication or acute hemolysis during infection. Our results differ from the largest report published so far from the US. Indeed, in 750 COVID‐19 cases of international SECURE‐SCD Registry, pain was the most common presenting symptom during COVID‐19 illness, with VOC in 67.4% and ACS in 28.5% of adults [16]. History of pain was a risk factor of hospitalization (RR, 1.8; *p* <0.002) and for serious COVID‐19 (RR 2.0; *p* = 0.02) in adults [16], but in this study, the authors categorized serious COVID‐19 illness when patient had pneumonia even without hypoxia. In addition, they presented evidences that HU treatment had no effect on hospitalization and COVID‐19 severity, but was associated with lower risk of presenting with pain in adults during COVID‐19 (RR, 0.9; *p* = 0.02) [16]. Overall favorable outcomes of our Guadeloupean series are also different from those reported by Minniti et al. [16] in SCD Afro‐American individuals for which VOC and ACS were observed in 65% and 50%, respectively, with 75% patients requiring hospitalization and 10.6% patients dying. Likewise, in our cohort, genotypes, diabetes, and BMI were not associated with hospitalization or death in this US study. In contrast, US patients with preexisting kidney disease were more likely to be hospitalized. Among inpatients, their results show that age, pulmonary hypertension, stroke, and cardiac or kidney disease were associated with death. Such associations were not detected in our cohort. However, nearly 30% (27%) of our patients had chronic kidney disease, but kidney injury was not associated in our cohort with COVID‐19 severity or hospitalization, or death. It is worthwhile to notice that the proportions of patients with medical history of stroke and cardiac or renal insufficiency were lower in our cohort compared to those reported in the US cohort, and there was no patient with pulmonary hypertension, a complication associated with the highest COVID‐19 severity in the US Study [16]. Unlike data reported by Arlet et al. [13], older age is not here associated with a worse prognosis. HU treatment, transfusion or exchange transfusion, and phlebotomy therapy that are related to a more severe SCD course, did not show any association with the severity or outcome of COVID‐19 in our series. The only patient who unfortunately died was in the low prognostic group, based on risk factors identified in general population.

Symptoms of COVID‐19 in our SCD patients do not differ from those of the general population [21], and VOCs or ACS presented by almost half of our patients were not particularly severe, and promptly resolved. Surprisingly, there were very few cases of SARS‐CoV‐2 pneumonia that probably partly explains this favorable evolution. Hospitalization was motivated by management of VOC, ACS, or worsening anemia and not by the severity of infectious episode. Simple/exchange transfusion in about 1/3 of the hospitalized patients could allow the rapid resolution of the symptoms related to their SCD and a favorable outcome. The survey of published papers written by Sayad et al. [22] shows similar results with 36.5% of SCD adults with COVID‐19 that required simple/exchange transfusion during hospitalization.

In series reported by Singh et al. who compared COVID‐19 in SCD individuals with matched black patients without SCD between January and September 2020 in USA, the former remained at higher risk of hospitalization (relative risk [RR], 2.0; 95% CI [1.5–2.7]) and development of pneumonia (RR, 2.4; 95% CI [1.6–3.4]); the case fatality rate for those with SCD compared to patients without SCD was no significantly different [23]. In a retrospective multicentric cohort study in Barhrain between February and July 2020, in whom 38 SCD patients were compared to a randomly select sample of non‐SCD patients with COVID‐19, SCD was not a risk factor for severe COVID‐19 outcomes in hospitalized patients [24]. The causes of discrepancy remain largely unknown and deserve further studies. However, it is tempting to speculate that the favorable outcome in our cohort could be related to the absence of comorbidity known to be linked to the more severe forms of COVID‐19, associated to early and coordinated management of the SCD patients.

We did not find any predictive factors for hospitalization of SCD patients except for probably protective effect of ACE inhibitor. All patients continued ACE inhibitor during infection. With the capability of inducing elevated expression of ACE2, the cellular receptor for SARS‐CoV‐2, ACE inhibitor treatment may have a controversial role in both facilitating virus infection and reducing pathogenic inflammation. The most recent studies tend to support continued use of ACE inhibitors during infection [25, 26], but, up to now, there was no study that had confirmed the potential benefits of ACE inhibitors for patients with COVID‐19.

SC patients, more frequently encountered in the French West Indies than in mainland France, represent here 37% of our cohort and unsurprisingly show a higher basal Hb level and BMI than SS patients. This percentage is similar to the proportion of SC patients in the Guadeloupean active file (38%). The only obese patients in our cohort were SC patients but their IMC was <35. Beside obesity, SS and SC groups are remarkably similar in terms of comorbidity and vaso‐occlusive events in the last 3 years. Unlike data published by Arlet et al. [14] that showed in multivariate analysis that the SC genotype was a strong independent risk factor for mechanical ventilation or death, there is no difference in terms of severity and outcome of COVID‐19 between SC and SS patients in our cohort. The only death occurred in an 85‐year‐old SC patient who had risk factors for poor prognostic. Our results related to SC genotype are also different from those of UK [15] series, where the proportion of patients who required critical care was higher in mild genotypes (SC, Sβ^+^‐thal, or SE) than severe genotypes (SS or Sβ^0^‐thal). Mortality was also higher in mild genotype group for which higher comorbidity factors were also more frequently encountered. In a previous series published by Paninpinto et al. in October 2020 [27], COVID‐19 deaths also occurred mainly in patients with mild SCD genotypes. These two series did not assess whether these findings were related partly by preexisting comorbidities or through association with other clinical, demographic, or socioeconomic risk factors. Overall, our data suggested that SC were not at higher risk of severe course of COVID‐19 than SS patients. However, our study is based on a limited number of symptomatic patients and thus may lack statistical power to identify significant differences between genotypes, or between inpatients and outpatients. Therefore, our finding needs to be confirmed on larger SCD cohorts.

## 5 CONCLUSION

Our Guadeloupean series surprisingly shows that SCD patients, regardless of their genotype, may exhibit a mild course with COVID‐19. Only 9.6% of active adult SCD patient file exhibited symptomatic SARS‐CoV‐2 infection that prompted a visit to our medical center. We currently do not know how many patients had asymptomatic CoVID‐19 and considering only these symptomatic patients would lead to an overestimation of the clinical signs during infection. Favorable outcome can be linked to the low prevalence of chronic end‐organ disease and severe occlusive disease in the last 3 years; the favorable outcome may also be related here to the absence of comorbidity known to be linked to the more severe forms of COVID‐19. Moreover, all our patients are regularly followed up both medically and socially in the same Reference Center relatively close to their home. This long‐standing coordinated management may partly explain this low prevalence of severe organ damage detected in our series and have undoubtedly facilitated prompt management of the COVID‐19 infection. Results of the different cohorts published in 2020 and 2021 continue to be contradictory; we must therefore continue to collect and analyze cases of SCD patients infected with SARS‐CoV‐2, with particular focus to SC genotype and to their frequent comorbidities, in order to identify the risk factors of severe COVID‐19 and ensure appropriate prevention and management. However, our experience has shown the benefits of early and close monitoring of the patients.

## CONFLICT OF INTEREST

The authors declare that there is no conflict of interest that could be perceived as prejudicing the impartiality of the research reported.

## AUTHORS’ CONTRIBUTION

Emmanuelle Bernit, Maryse Etienne‐Julan, Marc Romana, and Marie Dominique Hardy Dessources designed the study; Emmanuelle Bernit, Maryse Etienne‐Julan, Scylia Alexis‐Fardini, and Corine Charneau enrolled patients; Emmanuelle Bernit, Marie Dominique Hardy Dessources, Marc Romana, and Maryse Etienne‐Julan wrote the manuscript. Vanessa Tarer and Benoit Tressières performed the statistical analysis and discussed the data. All authors discussed the data, revised, and approved the manuscript.

## ETHICS STATEMENT

This study was approved by ethical research commission of the Centre Hospitalier Universitaire de la Guadeloupe (Registration number: A41_21_02_08_DREPA‐COVID).

## Data Availability

The data that support the findings of this study are available from the corresponding author upon reasonable request.
